# Nurses’ experiences of serving as a communication guide and supporting the implementation of a communication intervention in the intensive care unit

**DOI:** 10.1080/17482631.2021.1971598

**Published:** 2021-09-06

**Authors:** Anna Holm, Veronika Karlsson, Pia Dreyer

**Affiliations:** a Department of Anesthesiology and Intensive Care, Aarhus University Hospital, Aarhus N, Denmark; b Department of Health Sciences, University West, Trollhättan, Sweden; c Department of Public Health, Section of Nursing Science, Aarhus University, Aarhus C, Denmark; d Department of Global Public Health and Primary Care, University of Bergen, Bergen, Norway

**Keywords:** Augmentative and alternative communication, critical care, health communication, implementation science, intensive care unit, nurse-patient relations, evidence-based nursing, qualitative research

## Abstract

**Purpose:**

: To explore the experience of serving as a nurse communication guide, supporting the bottom-up implementation of a multi-component communication intervention prototype in the intensive care unit.

**Methods:**

: The overall frame was Complex Interventions, and the study was conducted within the phenomenological-hermeneutic tradition. Semi-structured telephone interviews were conducted with eight nurse communication guides. Data were analysed using a Ricoeur-inspired interpretation method.

**Results:**

: Two main themes emerged: 1) “The communication intervention components provided overview, a conceptual framework, awareness and room for reflection” and 2) “Being a communication guide illuminated the barriers and challenges of implementation”. Furthermore, a comprehensive understanding was established that illuminated experiences throughout the analysis: “An ICU communication intervention has to be adaptable to the specific situation and the double need for individualization but also provide overall guidance”.

**Conclusion:**

: Findings showed that as communication is inherent to all human beings, it can be difficult to change the communication behaviour of nurses. Therefore, a communication intervention in the intensive care unit must be sensitive to the nurse communication guides’ individual communication style. Furthermore, a communication intervention should provide nurse communication guides with overall guidance while at the same time remaining adaptable to the needs of each specific situation.

## Introduction

### Background

Nurse-patient communication in the intensive care unit (ICU) is a continuous, changeable, dyadic process that involves interchange of verbal and nonverbal information, needs and feelings. However, mechanically ventilated patients are deprived of the possibility to express themselves verbally and, for a period, only the nurse can communicate verbally during their encounter. The patient is trapped in a voiceless world where the expression of thoughts, wishes, needs and concerns depends on the nurse’s ability to interpret his or her nonverbal communication (Carroll, 2007; Danielis et al., 2020; Karlsson et al., 2012). Nurse-patient communication in the ICU is affected by multiple factors; 1) the patients’ communicative characteristics and general condition, which may vary from day to day; 2) the nurses’ communication approach and care focus; 3) the relation between the nurse and patient; and 4) external factors, e.g., pressure from busyness, the ICU environment and strategies implemented to promote communication (Dithole et al., 2016; Finke et al., 2008; Anna Holm et al., 2020). Difficult or failed communication is frustrating for nurses and patients alike (A. Holm & Dreyer, 2018). It has a negative effect on patients, emotionally as well as psychosocially (Guttormson et al., 2015; Khalaila et al., 2011; Koszalinski et al., 2020; Tembo et al., 2015). Moreover, it affects nurses’ experiences of professionalism and job satisfaction (Freeman-Sanderson et al., 2019; IJssennagger et al., 2018; Rodriguez et al., 2015). Several research syntheses have shown that numerous studies have been conducted to optimize communication for mechanically ventilated patients; they have also established that ICU nurses are the gatekeepers for this to succeed (Carruthers et al., 2017; Dithole et al., 2016; Anna Holm et al., 2020; Karlsen et al., 2019; ten Hoorn et al., 2016). However, implementation of interventions in clinical practice is known to be notoriously difficult (Hallberg & Richards, 2015), which is also the case in the ICU setting (Bjurling-Sjöberg et al., 2018; Bjurling‐Sjöberg et al., 2015; Handberg & Voss, 2018). In the ICU, advanced treatments like administering inotropes, managing dialysis treatment or mechanical ventilation and other technical procedures constitute a considerable part of the nurses’ caring tasks. Non-lifesaving focuses like communication may therefore unintentionally assume a low priority (Garrett et al., 2007; Handberg & Voss, 2018). In these cases, communication may become ‘non-caring’ (Karlsson et al., 2012). The SPEACS study tested the effect of a multi-component communication intervention in the ICU (Happ et al., 2014). This lead to an increase in the use of communication tools and lessened patients’ communication difficulties. Furthermore, it showed a positive effect on communication frequency and increased nurses’ positive communication behaviour significantly.

Implementation of new evidence-based guidelines or recommendations almost always implies that healthcare professionals need to change their behaviour, including their communication practices, to some degree (Colquhoun et al., 2017; Johnson & May, 2015). Implementation challenges may be countered by adoption of a specific implementation plan or strategy (Hallberg & Richards, 2015; May et al., 2016; Peters et al., 2013). Such a plan or strategy may be single- or multi-faceted and may include, e.g., education or actions like reminders or feedback (Johnson & May, 2015). Furthermore, evidence suggests that persuasive strategies embracing local opinion leaders may strengthen the implementation process (Holleman et al., 2014; Johnson & May, 2015). The assumption behind this is that interpersonal contact in clinical practice and empowerment via peers are key factors in changing healthcare professionals’ behaviour (Holleman et al., 2014).

To comply with potential implementation challenges, we introduced nurse communication guides as a novel approach for the bottom-up implementation of a communication intervention. The present study was conducted to gain insight into the communication guides’ experiences of implementing and working with a multi-component communication intervention prototype designed for the specific setting. This was done to be able to evaluate the intervention and the implementation strategy chosen. To our knowledge, no previous studies have focused on the experiences gained while supporting a bottom-up implementation of a communication intervention in the ICU.

## Methods

### Aim

The aim of this study was to explore the experience of a serving as a nurse communication guide, supporting the bottom-up implementation of a multi-component communication intervention prototype called the ICU-COM.

### Design

The overall study frame was the Medical Research Council’s (MRC) framework for developing complex interventions in healthcare (Craig et al., 2008). Within this frame, qualitative approaches are recommended to build an understanding of the experiences of the participants e.g., when developing or evaluating interventions (Fegran et al., 2014). We chose to conduct this current study within the phenomenological-hermeneutic tradition to develop an in-depth understanding of the nurses’ experiences of the phenomenon under exploration. The methodology was inspired by the French philosopher Paul Ricoeur. He is known for bridging phenomenological understanding with hermeneutic explanation, thereby bringing forward meaning in the form of comprehensive understanding (Ricoeur, 1984). This approach ensures strong coherence between data collection and data analysis. We conducted qualitative telephone interviews with an open approach to understand the phenomenon. The subsequent analysis comprised a movement between the parts and the whole in a hermeneutic spiral. To secure an explicit and complete reporting of the study, the Consolidated Criteria for Reporting Qualitative Research (COREQ) checklist was used as a guideline (Tong et al., 2007), see Supplementary File A.

### Intervention development and implementation

We developed a multi-component communication intervention prototype consisting of three components: 1) a communication strategy with an algorithm and the BASIS communication frame, which served as nurse support tools; 2) low- and high-tech communication tools; and 3) education of nurses in various situations. A detailed description of the intervention called the ICU-COM was published in a previous study (Anna Holm et al., 2021). We engaged local opinion leaders as a strategy to implement the ICU-COM during a test phase (from September 2021 to January 2021) during which the intervention’s feasibility and acceptability were evaluated. The local opinion leaders were called communication guides and 15 volunteered for the role. All communication guides participated in a four-hour workshop during which they were introduced to the project, the ICU-COM components and their role as communication guides. Subsequently, they worked in clinical practice serving as bottom-up supporters during implementation. We aimed to have the communication guides disseminate knowledge about the ICU-COM and serve as role models for their colleagues in order to enhance the intervention uptake.

### Participants

The participants in this study were nurses who volunteered to serve as communication guides (n = 15), making the sampling purposive. The only requirements for participation in the interview were a bachelor’s degree in nursing, employment at the participating ICUs during the study period and willingness to narrate one’s experiences. Therefore, the nurses enrolled had experience from a broad range of surgical and medical specialities within ICU nursing care.

### Setting

This study was conducted at an ICU department at a university hospital in Denmark. The ICU department has four smaller units; three general service and one neurological unit. The ICUs serve the needs of the most critically and acutely ill patients requiring highly specialized care and treatment. The ICU patient flow comprises approximately 4,000 patients per year. Approximately 60% of the nurses employed at the department are certified critical care nurses. The department follows national and international recommendations and therefore uses sedatives as little as possible (Devlin et al., 2018). The nurse-patient ratio is 1:1, and nurses are the main communication partners as they accompany the patients 24 hours a day.

### Data collection

Data were collected between January and March 2021 by the first author (AH), who has experience in research and clinical nursing in the ICU. The data collection method used was semi-structured telephone interviews (Brinkmann & Kvale, 2018). Originally, we intended to use focus group interviews. However, due to the COVID-19 pandemic, it was not possible to bring together several informants from different ICU sections in the same room. Consequently, we changed the design to comply with hygienic requirements and the restriction on gatherings of people at the time. All nurse communication guides (n = 15) were invited via email and received a participant information sheet with details about the study along with the invitation. The interviews were conducted whenever convenient for the participants. At the beginning of the interview, nurses were informed once more about the study to ensure that they understood the aim and how data would be used. The interviews were structured via an interview guide designed for the purpose. The first question was: “Can you tell me how you experienced being a communication guide during the project?”. The interview guide contained several questions elaborating on the experience and included follow-up questions allowing researchers to reach a deeper understanding. Data were recorded using a mobile phone app. Recordings were subsequently transcribed verbatim.

### Data analysis

The semi-structured interviews were transcribed and analysed using a Ricoeur-inspired interpretation method (Dreyer & Pedersen, 2009). Based on Ricoeur, fixating a narrative in text, e.g., via the transcription of an interview, is an important step of distanciation where objectification of the text may provide an understanding of what the text refers to (Ricoeur, 1973; Ricœur, 1976). This distanciation releases the text from the author making it a medium through which we can give it a life of its own and build a new understanding of the nurses’ lived experiences (Dreyer & Pedersen, 2009; Ricoeur, 1973). The analytic process of reaching this understanding comprised three steps: 1) The naïve reading during which the researchers establish an overall sense of the text as a whole. 2) The structural analysis that moves from what the text says to a deeper understanding of what it speaks about and, eventually, the development of themes. 3) The critical analysis and discussion during which a deeper understanding of the interpretation is reached by discussing the themes and any new perspectives from the literature. The analysis was conducted in Nvivo 12 where the text was coded by meaning. The systematic approach and use of Nvivo 12 allowed for a movement between the parts and the whole in a hermeneutic spiral, revealing the in-depth meaning of the data (Dreyer & Pedersen, 2009; Ricoeur, 1984). Table I is an illustration of how the themes were reached in the structural analysis.

**Table I.** Example of the structural analysis

What the text says	What the text speaks about	Theme
*“It makes good sense to have this pocket guide to consider whether I have included all aspects and possibilities.”*	Having a communication strategy that contains an algorithm and the BASIS frame meant that the nurse communication guides had tools supporting them in getting an overview.	**The communication intervention components provided overview, a conceptual framework, awareness and room for reflection**
*“You just become so happy when you succeed [laughs], and there are a lot of times when I don’t succeed, but when I do, it gives me energy that I ‘cracked the code’. And the strategy has definitely supported me in this, so that more often I’m able to interpret what the patient is trying to say.”*		
*“I think that what I was most surprised about and that I hadn’t given so much thought was the importance of how we document and describe how the patient communicates, what the different signals mean. I think that I have become more aware of this.”*	Nurses described that they became more focused on documenting their communication in detail, thereby allowing their colleagues to draw on their observations and experiences	
*“And to describe in detail, that communication with this patients works best this way, I think that I have become more focused on that.”*		
*“I have become very aware of how to have a systematic approach when I ask questions.”*	Others experienced that the project allowed them to develop new insights and techniques especially concerning systematism.	
*“I am more conscious about some things for example, how to have a systematic approach with the conscious patient”*		
*“I have become more attentive towards the fundamental principles that you need to consider. Does the patient have his hearing aid and glasses? Are we sure that we have a clear yes/no response? You know, all those things.”*	The nurses also described how the basic principles of communication had become more evident to them; e.g., giving the patients time to respond, providing them with glasses or hearing aids, securing a clear yes/no response and asking short and precise questions.	
*“I have become better at observing when others interact with the patients, especially giving the patient time to respond because they have latency”*		
*“Communication is an area that is hard to put into words, how I sense different things. And I actually thing that the algorithm helps me to do that”*	The nurses found that communication was a phenomenon that was difficult to put into words, and they did not possess as extensive a vocabulary as for other more instrumental or technical parts of nursing care, e.g., ventilator treatment. Being a nurse communication guide increased their vocabulary and ability to discuss nurse-patient communication with students and colleagues	
*“I’ve become more focused about the subject and I’ve been able to have conversations with my colleagues and bring it into play in our group.”*		
*“It will be much easier for me to bring it up, because I can base my guidance on this [the algorithm and low-tech communication book]. I can show them that we have these categories to work with”*	Furthermore, the intervention provided tangible tools to apply when supervising newly employed nurses or students.	
*“We have to remember that many experienced nurses supervise younger nurses, and then it is nice to have something to show them and make them reflect upon.*		
*“I have become more conscious about the importance of communication and that I actually have many options to draw on. I have also become more reflective about others’ communication and can distinguish between what was a good approach and what wasn’t quite as good [laughs]. So more awareness”*	The nurse communication guides described that they were able to reflect upon communication at a deeper level	

### Ethical considerations

According to Danish law, interview studies do not require ethical approval from the National Ethics Committee. The leading staff of the ICU departments approved the study. Nurses received details about the study via an email. An information sheet was attached describing aspects of confidentiality and informed consent. Participation was completely voluntary. Identities were anonymized using a coding system, and all data were kept safe in a server at the hospital. Furthermore, nurses were instructed to give verbal consent at the beginning of the telephone interview, which was then transcribed. The study followed the principles of the Declaration of Helsinki.

## Findings

### Interviews and participants

Eight nurses volunteered to participate in the interviews; their overall characteristics are presented in Table I. The interviews lasted 20–35 minutes. (Table II)

**Table II.** Characteristics of the enrolled nurse communication guides

Nurse	A	B	C	D	E	F	G	H
Age	25	60	29	53	58	42	39	38
Gender	F	F	F	F	F	F	F	F
Registered nurse (year)	2019	1988	2017	1993	1988	2002	2005	2009
Years of ICU experience	2	30	4	26	28	16	14	12
Degree in critical care nursing	No	Yes	No	Yes	Yes	Yes	Yes	Yes
Special function¶	No	No	No	Yes	Yes	Yes	Yes	Yes
Employment at ICU section	B	A	A	A	C	D	B	D

= Nurses who poses a special function in clinical practice e.g., responsible for introduction and training of newly employed nurses, responsible for development and implementation of new initiatives in clinical practice or coordinator of continuity in patient care

We established two themes and an overall comprehensive understanding, illuminating experiences that were present throughout the findings.

### Naïve reading

Overall, the nurses described their experiences of being a communication guide in relation to their role and function in the project. Implementation successes and barriers are illuminated. Particular attention was given to the challenges associated with linking the theoretical knowledge obtained during the workshop to bedside patient care. Furthermore, the nurses described that their own communication was strengthened and supported. This was for example, because they gained a systematic approach and became more aware of the importance of communication. Also, they established and extended the vocabulary needed to discuss the subject with colleagues and students and secure sufficient documentation of their interaction with patients. Furthermore, the naïve reading showed how the nurses’ personal communication style affected their use of the ICU-COM and established that the communication intervention had to accommodate a wide range of needs.

### Structural analysis

**The communication intervention components provided overview, a conceptual framework, awareness and room for reflection**

The nurse communication guides described how their own communication was strengthened and supported by their project participation. Having a communication strategy that contained an algorithm and the BASIS frame meant that the nurse communication guides had tools supporting them in getting an overview: “It makes good sense to have this pocket guide to consider whether I have included all aspects and possibilities” (D). Furthermore, nurses described that they became more focused on documenting their communication in detail. This allowed their colleagues to draw on their observations and experiences.

The nurses’ communication did not change completely. However, being a communication guide meant that they made changes based on their preferences, professional style of communication, knowledge and previous patient experiences combined with the knowledge they obtained from the workshop and e-learning course. It was described how a nurse already had her own integrated systematic approach before the intervention: “I’m not sure that I communicate differently from what I did previously. I use these pictograms that are divided into different categories, but I already did that previously” (H). However, participation in the workshop and implementation of the intervention meant that she was not alone with this approach; she could give words to a skill that she had acquired over the years so that others could be inspired and enlightened.

The nurses found that communication was a phenomenon that was difficult to put into words. They did not possess as extensive a vocabulary as for other more instrumental or technical parts of nursing care, e.g., ventilator treatment. However, being a nurse communication guide increased their vocabulary and ability to discuss nurse-patient communication with students and colleagues: “I’ve become more focused about the subject and I’ve been able to have conversations with my colleagues and bring it into play in our group” (F). Thus, being a communication guide increased their conceptual framework for communication with mechanically ventilated patients.

The nurse communication guides described that they were able to reflect upon communication at a deeper level: “I have become more conscious about the importance of communication and that I actually have many options to draw on. I have also become more reflective about others’ communication and can distinguish between what was a good approach and what wasn’t quite as good [laughs]. So more awareness” (G). Others experienced that the project allowed them to develop new insights and techniques especially concerning systematism: “I’ve become really aware about having a systematic approach when I ask questions” (G). Also, knowledge about the importance of being patient-centred made nurses more conscious about considering all needs—physical, emotional and social—when asking yes/no questions: “To use it [pictograms] and take into account if the patient is actually wanting to say something completely different” (H). The nurses also described how the basic principles of communication had become more evident to them. For example giving the patients time to respond, providing them with glasses or hearing aids, securing a clear yes/no response and asking short and precise questions. Furthermore, the intervention provided tangible tools to apply when supervising newly employed nurses or students: “It will be much easier for me to bring it up, because I can base my guidance on this [the algorithm and low-tech communication book]. I can show them that we have these categories to work with” (H). Thus, the intervention could create a room for reflection and discussion among nurses with various level of experience.

### Being a communication guide illuminated the barriers and challenges of implementation

Testing the intervention prototype gave the communication guides insight into the challenges of implementation. The study period was especially busy due to the COVID-19 pandemic; the hospitals were under pressure and bed occupancy rates were high. This was experienced as: “COVID stole the picture” (E). However, the nurse communication guides also described implementation challenges that would also have been experienced in a normal period. Other competing quality improvement projects affected the implementation. Among others, the nurses described that they had to respond to many interventions or guidelines and prioritize where to invest their energy: “There are always new initiatives and you have to consider what is important to focus on” (D).

Another barrier described were nurses who had extensive experience: “Actually, many years of experience in the ICU may be considered a barrier” (F). This group was described as having settled so firmly into their role as critical care nurses that it was difficult to change their behaviour and way of practising nursing care. The communication guides found that nurses with little or few years of experience were more adaptable. They were more open to new ideas and more minded towards taking in the knowledge and principles of the ICU-COM: “Many newly qualified nurses are employed in the ICU, and at that point you are completely open and ready to receive new input. If I had been introduced to it at that point, I could have made it into a habit from the beginning” (A). Therefore, the communication guides emphasized the importance of the intervention being presented during the introductory programme for new nurse employees.

One dominating implementation challenge was bridging the gap between knowledge and practise: “There is a big difference between presenting something theoretically and being close to clinical practice. To find a patient and test it so that it becomes meaningful at that moment. It is probably those experiences that will help make it successful” (G). Also, this is where the communication guides had the opportunity to bring the intervention into play: “It is probably at that point that we as communication guides could have made a bigger effort to succeed” (G). Linking the knowledge with patient cases would make the intervention less abstract and more clinically meaningful. However, this was occasionally considered difficult and illustrates how practice was more complex than theory.

Furthermore, it took courage to stand out and be the role models who lead the way: “I feel that it can be difficult to direct the attention towards it” (D). Also, it could be difficult to remember to use the communication intervention and find the excess energy to identify the unique examples that served to link theoretical knowledge to practising communication bedside.

## Comprehensive understanding—An ICU communication intervention has to be adaptable to the specific situation and the double need for individualization but also provide overall guidance

Across the findings, the analysis revealed a comprehensive understanding showing that a nurse-patient communication intervention in the ICU needs to accommodate many different and individual needs. This was both with respect to contents, design and implementation strategy. The four ICU departments had different cultures and routines, and even small differences affected how the test phase proceeded and how the communication guides were able to support implementation. The participants highlighted that besides differences in patient categories, it was difficult to pinpoint where in the exact difference lied. However, they stressed that each ICU department was unique: “We’re kind of special” (B).

Furthermore, findings showed how the nurses experienced that ICU patients were a heterogeneous group with very diverse needs, communication competences and characteristics affecting communication. The communication guides described that it was important for the ICU-COM to accommodate this diversity. Therefore, they were constantly vigilant to their patients’ continuously changing conditions and needs: “About the communication tools, I think that we have almost no patients where you can say that a specific approach works all the time. I really like the mindset that ‘what works now may not work tomorrow; perhaps nothing works then, but in the afternoon it might’. That you should not be so one-track minded and locked in your way of thinking. You need to see it as a variable, as something that can vary a lot even in the course of a shift” (F).

Besides the ICU departments and patients having different needs, the nurse communication guides also described that they themselves had individual needs, experiences and communication styles which affected their use of the ICU-COM and their role as a communication guide. This perspective was illustrated using the clinical guideline pocket books, which all nurses carry in their uniform, as an example: “It is hard to decide what should be in the pocket book, and it is the same for communication. What makes sense to people?! Hence, some of the things in the pocket book would not make sense to more experienced nurses but would be really important for newly employed nurses. They do not know things like the back of their hand yet and they would look at the algorithm. And then later it becomes more personalized, you find your own way of doing things, you get information from different places that you use as inspiration and then you find a style that fits you. But you do not have that in the beginning.” (H). The quote shows that the nurses’ levels of experience differ, thereby affecting their need for support tools e.g., an algorithm as provided in the ICU-COM. However, this finding also encompassed the aspect of what came natural to them and how their professional communication style came into play: “I think that we all have different ways of doing it and different approaches “ (E). The nurse communication guide elaborated on the previous statement by comparing the high-tech and the low-tech tool: “It is not because there is something wrong with how the app works, I think that it is really good. But I just do not know when I should choose the app in favour of the low-tech communication book. It just doesn’t come natural to me to go out and get a tablet” (H). Throughout the findings, the nurse communication guides each described different aspects of the communication intervention that they had used depending on what made sense to them. Furthermore, they described that it was important for the intervention to be flexible and capable of meeting their individual needs: “One great thing about this project is that it doesn’t order me to take a specific approach, but that we are presented to different options” (G).

**Figure 1.**
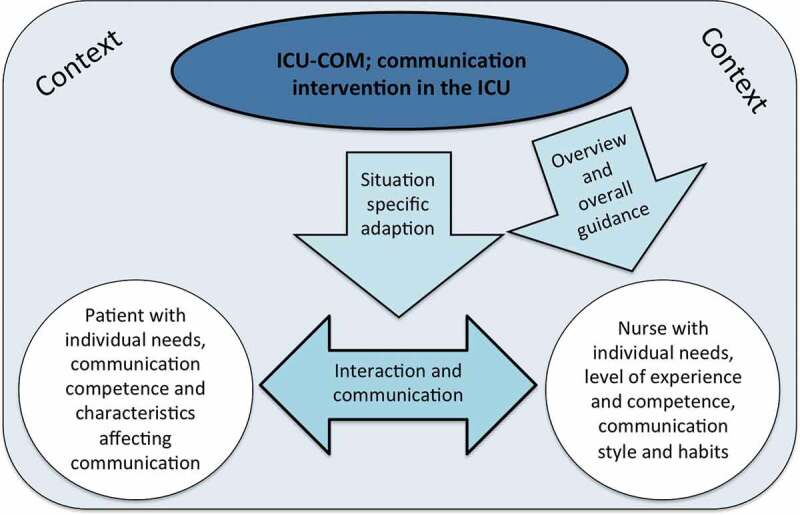
The comprehensive understanding shows how the nurse communication guide finds that the ICU-COM needs to provide overall guidance for their work while at the same time being adaptable to the specific situation and the interaction between the nurse and the patient. The context affects both the patient and the nurse and it determines, in part, how it should be designed and implemented

Overall, the findings showed that in the nurse-patient relation, a double need existed for the intervention to be individualized: “There needs to be something for all types of patients and all types of nurses, and then you can choose what makes sense to you” (H). Thus, our findings revealed that a communication intervention in the ICU needs to be adaptable both to the context and the nurse-patient interaction and the communication competencies and needs of both of them. On the other hand, the nurse communication guides also described the advantage of having an overall strategy that helps establish an overview and guidance in the form of a systematic approach, assessment and documentation: “Where I know with this strategy, I’ll figure out what the problem is” (E). One example of useful guidance was a questionnaire technique with overall categories that was consistent to patients. This made it easier to recognize the approach and patients could be confident that the nurse would get to the question that was meaningful to them: “It’s how you use your yes/no questioning technique instead of the patient being dependent on which nurse it is” (C). In line with the above statements, the comprehensive understanding underpinned two important aspects. First, that a communication intervention in the ICU had to strike a balance between providing overall guidance and being adaptable to the specific situation. This allowed the nurse to incorporate individual competencies, habits, experiences and styles of communication. Second, it had to take into consideration the individual patients’ characteristics, background and needs affecting communication. Furthermore, the context affected all aspects of a communication intervention in the ICU. The comprehensive understanding established in the study is summarized in Figure 1.

## Critical analysis and discussion

The two themes and the comprehensive understanding identified in the structural analysis will now be discussed by including relevant literature and thereby lift the results to a more general understanding. The analysis shows how nurses experienced assuming the role as communication guides. This was challenging in some respects, as it drew on their energy and called for them to stand out, be a role model and influence the way that their colleagues practiced communication with mechanically ventilated patients. However, their experiences also triggered reflection and awareness about their own nursing practice and about what constitutes good communication. Furthermore, working with the intervention gave them an overall conceptual framework and tangible tools with which to support their own communication, supervision of students and discussions of the subject with colleagues.

The comprehensive understanding showed that a communication intervention in the ICU is context dependent and has to take the many needs of the nurses and the patients into account. The present study was limited to the nurse-patient perspective. Therefore, other healthcare professionals may have other needs that also need to be taken into account in the development and implementation of a communication intervention in the ICU (Garrett et al., 2007). It is well known that ICU patients are a heterogeneous patient group and that their specific backgrounds, characteristics and individual needs affect communication (A. Holm & Dreyer, 2018). The present study adds to this knowledge because the group of nurses working it the ICU also form a heterogeneous group with respect to communication as they represent different communicative preferences and styles. A previous study described a similar finding as an intervention made the participants more aware of their personal nursing practice and established that not all nurses shared a common practice in relation to communication with mechanically ventilated patients (Noguchi et al., 2018). Other studies found challenges related to optimizing communication in the ICU. These challenges included the nurses’ behaviour or attitude towards augmentative and alternative communication (AAC) as well as other barriers, e.g., time constraints, patient characteristics, uncertainty about the use of or overly complicated strategies and tools (Handberg & Voss, 2018; Radtke et al., 2012). The findings of the present study may help explain why it can be so difficult to change nurses’ attitudes or behaviours in relation to patient communication; communication is inherent to human beings. Changing communication in a professional context may be difficult because the nurse then needs to change his or her personal trait or style of communication. From a phenomenological perspective, changing the nurses’ communication style or approach may be seen as a disturbance of the ‘lived body’ or the ‘body of personal experience’ (Thomas, 2005). Merleau-Ponty described that when there is a disturbance between the body and the world, the persons’ existence may be deeply shaken (Thomas, 2005). In the case of the communication guides, the nurses may unconsciously experience that changing their professional communication also entails changing a part of their body of personal experience. This may be perceived as an indirect threat to their existence. In the study by Handberg and Voss (2018), the authors found that even though communication was important for the nurses’ caring ontology, and despite the fact that they were motivated for and enthusiastic about AAC, they “mentioned how strong the force of habit was” (p. 109). Hence, changing their routines and the communication culture proved difficult. Again, perspectives from Merleau-Ponty may elaborate this point as he explained that there is no distinct separation between ‘bodily conduct and intelligent conduct’. Rather, we have an embodied consciousness (Moya, 2014). With reference to Merleau-Ponty, Moya (2014) stated that: “In habits, the body adapts to the intended meaning thus giving itself a form of embodied consciousness” (p. 1). In our findings, the nurse communication guides had an embodied consciousness affecting their habits and behaviour in relation to communication. Therefore, it seems important to consider carefully how to work with nurse behaviour change when implementing single- or multi-level communication interventions in the ICU. The behaviour change wheel may be utilized to support this process (Michie et al., 2014).

Our findings indicate that the communication routines and habits need to be embedded in the nurses early in their career; it is more difficult to change the behaviour of experienced than newly educated nurses. Studies show that all nurses may experience communication difficulties with intubated patients irrespective of their level of experience (Jansson et al., 2019; Karlsson & Bergbom, 2015; Mortensen et al., 2019; Rodriguez et al., 2015). However, it remains unknown if a difference exists in the ease of behaviour change in relation to patient communication for novices, competent or expert nurses in the ICU. Furthermore, local circumstances may determine what the curriculum of newly employed nurses should comprise and how they should be trained. Garrett et al. (2007) suggest workshops with Power Point presentations, role-play exercises, discussions and story-telling activities as well as casework. Another study highlighted that the patients’ experiences can be used as persuasive arguments and a motivational factor for the nurses in relation to communication (Noguchi et al., 2018). Focus on role-playing exercises based on realistic patient cases may enhance clinical relevance and boost nurses’ willingness to adhere to educational courses aiming to increase their knowledge and communication skills. The SPEACS-2 provides strategies, tools and an educational course for critical care (Happ, 2013; Trotta et al., 2020). However, language and cultural differences may be a barrier to translating this into other settings. To achieve a deeper understanding of these barriers, future studies should focus on the application of an overall evidence-based educational and training course as well as on the curriculum in various settings.

Our findings show that a major challenge for the communication guides was to integrate the theory they learned at the four-hour communication workshops into their bedside nursing practice. Specifically, they found that it was difficult to pass knowledge on to their colleagues in a manner that ensured that they fully understood the principles behind the ICU-COM. Evidently, practice is more complex than theory. The theory-practice gap is well known in nursing, and the challenge of incorporating theory and practice is documented in a broad range of settings (Leach & Tucker, 2018). In the ICU setting, teaching strategies such as guided reflection are suggested as a way to support critical care trainees in using theory when working in close relation with the patient (De Swardt et al., 2012). A similar strategy could have been utilized in the communication workshop of the ICU-COM in the present study. This would have provided the nurse communication guides with more tangible tools to underpin the transformation of theoretical knowledge into bedside patient care. Furthermore, the nurse communication guides suggested that they could have brought up specific patient cases in their discussions with colleagues when this made sense in clinical practice. Contextual and situational aspects, e.g., the social, structural and cultural perspectives, affect how evidence-based practice and research are translated into day-to-day nursing care (Leach & Tucker, 2018). Therefore, it seems vital to map these aspects before choosing an implementation strategy. This should include the important aspect of bridging the knowledge-practice gap when working with optimization of nurse-patient communication in the ICU. Our findings also show that researchers and clinicians who want to work with nurse-patient communication interventions need to be aware that small stepwise changes rather than major leaps may be considered a success within this area. Also, implementation is a lengthy process and this should be taken into consideration during planning.

Furthermore, our findings touch upon the subject of clinically meaningful interventions, as the nurse communication guides described how important it is that the intervention makes sense to the individual nurse. They also highlighted how the intervention needs to be flexible to underpin their professional communication preferences and style, while at the same time providing them with overall guidance. Nursing interventions are typically complex, and this needs to be taken into account when designing, evaluating and implementing the interventions (Fegran et al., 2014). A variety of implementation strategies are available that may be either involuntary (e.g., laws or regulations) or voluntary. The latter may focus on extrinsic or intrinsic motivation (Hallberg & Richards, 2015, p. 283). In this study, our approach focused on intrinsic motivation. This was primarily done by trying to enhance the nurse communication guides’ competences and attitudes via the training and instruction provided at the workshop, but also by sending reminders via weekly newsletters making the approach somewhat behaviour oriented. Our findings highlight that the nurse communication guides appreciated the voluntary implementation strategy. We therefore found that a subject of relational and interpersonal nature needs to allow nurses to bring their own lived experiences and preferences into the communication to ensure that the intervention is meaningful to them and works in clinical practice.

## Strengths and weaknesses

Due to the COVID-19 pandemic, clinical practice was under considerable stress when the study was conducted. Hence, nurses had very little energy and time to participate in the interviews. As a result, it was difficult to recruit nurses for the interviews and only eight were enrolled. However, we believe that the informants illuminated the explored phenomenon, providing thick in-depth descriptions. The data were dense and rich with lengthy comments, indicating a good data quality (Brinkmann & Kvale, 2018). As described in the findings, the COVID-19 pandemic also affected the overall project, because nurse communication guides had little time and energy to insist and press on with the implementation; COVID-19 simply dominated many aspects of the nurses’ personal and professional lives during the study period. This may have affected how the nurses handled their role as communication guides. If the study been conducted at another time, some of their experiences might have been different. The COVID-19 situation also meant that the original data collection design had to be changed from focus group interviews to individual telephone interviews. This means that the data obtained illuminate the experiences of the individual nurses, whereas focus group interviews would have captured perspectives and opinions that might have emerged from the interaction and discussions within the group (Brinkmann & Kvale, 2018). However, the individual telephone interviews may also have contributed to the nurses being willing to narrate more openly about their experiences. The telephone interviews meant that the interviewer and interviewee were not interacting face-to-face. Therefore, nonverbal communication could not be observed, and the interviewer had to be sensitive to noticing if the interviewee indirectly expressed something that was important for data or if she was uncomfortable sharing certain perspectives. Whether the data generation and quality would have been different if the intended focus group interviews had been conducted remains unknown. The analytical approach chosen was excellent in bringing out in-depth meaning and ensuring that the lived experiences of the communication guides emerged. Trustworthiness and rigour were addressed throughout the data collection, analysis and drafting of the manuscript as we sought be systematic and thorough, enhance confidence in the interpretation by providing quotes to bring out the findings, discuss the interview guide and analysis in the research team and broaden the findings in the critical analysis and discussion by including relevant literature. It remains uncertain if the data can be transferred to other settings. However, some aspects of ICU nursing care are unique to Denmark and Scandinavia and is not shared by nurses in other countries. This is for example a nurse-patient ratio of 1:1 and minimal use of sedatives (which is possible owing to the nurse-patient ratio). It remains uncertain whether other countries would be able to conduct a similar study. Even so, our findings might provide perspectives on the design of an implementation strategy for a communication intervention with mechanically ventilated patients.

## Conclusion

This study contributed with insights into the context/intervention interface as experienced by the communication guides. The first theme illustrates how working with the components of the ICU-COM intervention provided 1) overview, 2) a conceptual framework, 3) awareness and 4) a room for reflection. The second theme shows that the implementation process was challenging and had barriers relating to 1) the COVID-19 pandemic, 2) other competing quality improvement projects, 3) changing the behaviour of experienced nurses, 4) bridging the theory-practice gap and 5) having the courage to be role models for their colleagues.

Furthermore, our findings highlight that communication is inherent to human existence; from the second we are born, we communicate. Additionally, our personal communication style is adjusted throughout our lives, and as nurses, we develop a professional approach to communication. This approach is then adjusted based on our experiences—which patients we have encountered, which approaches have worked for us in the past, which theoretical knowledge we have obtained and which role models we have encountered on the way. The findings of this study show how this affects the nurses’ experiences of serving as a communication guide as their communication was altered to some degree, but they did not completely change their style of communication. The nurses may experience that a change of professional communication entails a change in their body of personal experience, which may unconsciously be perceived as a threat to their existence. This provides an explanation to why it can be difficult to change the communication behaviour of nurses. Therefore, they picked out the parts of the ICU-COM that were meaningful to them and incorporated the knowledge they gained at the workshop to the extent possible. Hence, it was important that the intervention balanced between being flexible in order to accommodate the individual needs and the specific situation while at the same time providing overall guidance without dictating in detail how the nurses should communicate with the mechanically ventilated patient.

## Relevance to clinical practice

The study contributed perspectives of relevance to clinical practice, specifically to the implementation of communication interventions in the ICU. Local opinion leaders such as communication guides may serve to support a bottom-up implementation strategy as they become role models who motivate their colleagues to utilize a range of strategies, tools or devices that may enhance good and effective nurse-patient communication. However, it is important to equip communication guides with tools and guide them on how to bridge the theory-practice gap, e.g., through guided reflection. Furthermore, clinical practice settings should be aware that it may take a long time to change the communication behaviour of nurses because professional communication preferences and styles are based on personal communication traits that have evolved throughout the nurses’ lives. For a communication intervention to be meaningful in clinical practice, it needs to provide overall guidance and be adaptable to the specific situation and to the nurses’ communication style. Our findings indicate that the implementation strategy should be inclusive and sensitive to the voluntary perspectives and avoid dictating in detail how the nurse should act. Additionally, clinical practice settings should lower expectations concerning the degree to which nurses may comply with the intervention and establish realistic evaluation goals according to which small alterations may also be considered a success.

## Supplementary Material

Supplemental Material
