# Supplementary material for: Nurses’ experiences of serving as a communication guide and supporting the implementation of a communication intervention in the intensive care unit
Source: Int J Qual Stud Health Well-being. 2021 Sep 6;16(1):1971598. doi: 10.1080/17482631.2021.1971598 (PMC8425701; doi:10.1080/17482631.2021.1971598)
Supplement: Supplemental Material [file ZQHW_A_1971598_SM0147.docx]

**Supplementary File A: COREQ checklist**

Domain 1: Research team and reflexivity

Personal characteristics

1. Interviewer/facilitator: Which author/s conducted the interview or focus group?

The first author, AH (page 7).

1. Credentials: What were the researcher’s credentials? E.g. PhD, MD

MScN, PhD student.

MScN, PhD, Senior Lecturer.

MScN, Professor of Nursing (title page).

1. Occupation: What was their occupation at the time of the study?

PhD student.

Senior Lecturer.

Professor of Nursing (title page).

1. Gender: Was the researcher male or female?

Female (title page).

1. Experience and training: What experience or training did the researcher have?

All authors are experienced nurses within intensive care and have conducted interview studies in this setting with both healthcare professionals and patients.

The second and last authors are senior researchers (title page).

Relationship with participants

1. Relationship established: Was a relationship established prior to study commencement?

Yes.

1. Participant knowledge of the Interviewer: What did the participants know about the researcher? e.g. personal goals, reasons for doing the research.

All participants knew the project leader (AH) from communication workshops and participation in the project.

1. Interviewer characteristics: What characteristics were reported about the interviewer/facilitator? e.g. bias, assumptions, reasons and interests in the research topic.

No characteristics were reported about the interviewer.

Domain 2: Study design (see page 6-10 in the main manuscript)

Theoretical framework

1. Methodological orientation and Theory: What methodological orientation was stated to underpin the study? e.g. grounded theory, discourse analysis, ethnography, phenomenology, content analysis, participant selection.

The overall frame was Complex Interventions and the data were collected within the phenomenological-hermeneutic tradition (page 6).

1. Sampling: How were participants selected? e.g. purposive, convenience, consecutive, snowball.

Purposive, all nurses who had worked as a communication guide were invited to participate in the study (page 8).

1. Method of approach: How were participants approached? e.g. face-to-face, telephone, mail, email.

Participants were invited via an email with an attached study information sheet (page 9).

1. Sample size: How many participants were in the study?

Eight (page 10).

1. Non-participation: How many people refused to participate or dropped out? Reasons? Setting

15 were invited and eight volunteered (page 8+10).

1. Setting of data collection: Where was the data collected? e.g. home, clinic, workplace.

The interviews were telephone interview where the participants were phoned at a time of their choosing, location unknown (page 8-9).

1. Presence of non-participants: Was anyone else present besides the participants and researchers?

No.

1. Description of sample: What are the important characteristics of the sample? e.g. demographic data, date, data collection.

Nurses who had worked as communication guides during the test phase of the intervention (page 10, table 2).

1. Interview guide: Were questions, prompts, guides provided by the authors? Was it pilot tested?

The interview guide was designed by first author and discussed with last author, it was not pilot tested (page 9).

1. Repeat interviews: Were repeat interviews carried out? If yes, how many?

None.

1. Audio/visual recording: Did the research use audio or visual recording to collect the data?

Audio via an telephone app (page 9).

1. Field notes: Were field notes made during and/or after the interview or focus group?

No.

1. Duration: What was the duration of the interviews or focus group?

20-35 min. (page 10).

1. Data saturation: Was data saturation discussed?

No.

1. Transcripts returned: Were transcripts returned to participants for comment and/or correction?

No.

Domain 3: Analysis and findings

Data analysis

1. Number of data coders: How many data coders coded the data?

One, first Author AH. These were discussed with last author.

1. Description of the coding tree: Did authors provide a description of the coding tree?

No.

1. Derivation of themes: Were themes identified in advance or derived from the data?

Derived from data.

1. Software: What software, if applicable, was used to manage the data?

NVivo 12 (page 10).

1. Participant checking: Did participants provide feedback on the findings?

No.

Reporting

1. Quotations presented: Were participant quotations presented to illustrate the themes / findings? Was each quotation identified? e.g. participant number.

Yes (page 11-17).

1. Data and findings consistent: Was there consistency between the data presented and the findings?

Yes (page 11-17).

1. Clarity of major themes: Were major themes clearly presented in the findings?

Yes (page 11-17).

1. Clarity of minor themes: Is there a description of diverse cases or discussion of minor themes?

No minor themes.
